# Thermotolerance screening of *Brassica carinata* genotypes using *in vitro* seed germination assay

**DOI:** 10.1016/j.heliyon.2024.e41113

**Published:** 2024-12-10

**Authors:** Leelawattie Persaud, Naflath Thenveettil, Ramdeo Seepaul, Bisoondat Macoon, Krishna N. Reddy, Kambham Raja Reddy

**Affiliations:** a Department of Plant and Soil Sciences, 117 Dorman Hall, Box 9555, Mississippi State University, Mississippi State, MS, 39762, USA; b University of Florida, North Florida Research and Education Center, Quincy, FL, 32351, USA; c United States Department of Agriculture-National Institute of Food and Agriculture, Lawrenceville, GA, 30043, USA; d USDA-ARS, Crop Production Systems Research Unit, 141 Experiment Station Road, P.O. Box 350, Stoneville, MS, 38776, USA

**Keywords:** *Brassica carinata*, Seed germination, Seed germination rate, High-temperature tolerance, Low-temperature tolerance

## Abstract

Temperature is a fundamental factor influencing the processes of seed germination. Investigating the response of carinata to thermal stress and establishing a dependable and efficient method for screening thermotolerance will enhance breeding programs and model applications. We assessed the response of 12 carinata genotypes to a range of eight temperatures, spanning from 8 to 37 °C, throughout the germination process. A four-parameter Weibull function effectively characterized the seed germination time course across various temperatures and genotypes. Quadratic functions effectively characterize the maximum seed germination and the rate of seed germination as a function of temperature across various genotypes. The average cardinal temperatures recorded were −0.14, 20.41, and 40.70 °C for maximum seed germination, while the temperatures for seed germination rate were 5.30 °C, 24.51 °C, and 43.71 °C, corresponding to minimum, optimum, and maximum conditions, respectively. A notable variation was observed in maximum seed germination, seed germination rate, and cardinal temperatures across different genotypes. The specified parameters were employed to screen the genotypes for their thermotolerance capabilities. The analysis of principal components and the cumulative response indices effectively characterized carinata genotypes for high- and low-temperature tolerance exhibiting distinct characteristics. The AX17004 was identified as a genotype with significant tolerance to high temperatures, whereas AX17009 demonstrated remarkable tolerance to low temperatures. In contrast, AX17002 exhibited sensitivity to both high and low-temperature stress. According to the classification of breed types, both double haploid and hybrid groups revealed consistent thermotolerance responses during the germination phase. In contrast, the inbred group exhibited a wider response cluster to both minimum and maximum temperatures. The *in vitro* assay method presents a cost-effective approach for evaluating thermotolerance in carinata genotypes.

## Highlights

• Twelve carinata genotypes were subjected to an *in vitro* thermotolerance screening at temperatures ranging from 8 to 37 °C.
• The seed germination time course was best described by a 4-parameter Weibull function.
• Maximum seed germination and seed germination rate showed quadratic functions against temperatures.
• Genotypes varied in their response to temperatures.
• Among 12 genotypes, AX17004 and AX17009 were identified as high- and low-temperature tolerant lines, respectively.

## 1 Introduction

Successful biofuel feedstock crops necessitate a high germination rate, emergence, and stand establishment [1]. The efficient establishment of new feedstock species in a novel environment relies on its capacity to emerge and establish uniformly and rapidly [2], as well as to yield under diverse environmental conditions. Climate change is progressing rapidly, resulting in variations in abiotic stresses, including frequent flooding, extended drought periods, and increased occurrences of both low and high-temperature episodes [3]. Hence, identifying adaptable genotypes and appropriate management practices is essential for optimizing yield [4,5]. The establishment of a crop stand is influenced by internal factors such as seed viability, maturation, genotype, and dormancy, as well as external factors, including water, light, temperature, and oxygen [6,7].

*Brassica carinata* A. Braun, known as Ethiopian mustard or carinata, is an oilseed crop recognized for its potential in biofuel production [8], attributed to its high erucic acid content [8,9]. Carinata, due to its area of origin, exhibits superior adaptation to semi-arid regions [10,11], demonstrating enhanced tolerance to moisture stress, elevated temperatures [12], seed shattering [13], drought [14], and diseases [15]. Carinata is the product of interspecific hybridization between black mustard (*B. nigra* L.) and wild cabbage (*B. oleraceae* L.), both of which are genetically related to *Brassica* species [16]. Carinata is an amphidiploid demonstrating high survival and adaptability [[17], [18], [19]] yet exhibiting low cold tolerance [20]. Genetic characteristics among genotypes may influence the adaptability range of a genotype to specific regions [21]. The Southeast Partnership for Advanced Renewables from Carinata (SPARC), led by the University of Florida, FL, USA, is engaged in efforts to eliminate physical, environmental, social, and economic barriers to its adoption and output while also addressing risks across the supply chain [22].

Temperature significantly influences seed germination, emergence, growth, and development [23,24]. It affects both the percentage of seed germination and the germination rate in most crop species [[25], [26], [27]]. Elevated temperatures can lead to restricted availability of photosynthetic assimilates during seed development [[28], [29]], potentially causing physiological damage and resulting in either lower or no germination [30]. Successful establishment of commercial crops requires knowledge of optimum growing conditions and effective establishment techniques [31,32]. In addition, the environmental conditions during growth and developmental stages influence the yield of crops, including *Brassica* species [33].

The process of seed germination typically occurs within a defined temperature range known as cardinal temperature [34,35]. This range includes minimum (T_min_), optimum (T_opt_), and maximum (T_max_) temperatures, which are essential for constructing models that predict seed germination and developmental processes [27,36]. Various mathematical models have established the correlation between germination rate and temperature [37,38]. For instance, quadratic, linear, and nonlinear regression models were employed to quantify the response of seed germination to temperature stress [[39], [40], [41], [42]]. However, the estimation of germination curves based on nonlinear regression leads to inappropriate standard errors, instead a log-logistic function will better fit the time-to-event data [43,44]. To date, no attempts have been made to document the seed germination and its cardinal temperatures in carinata species.

The assessment of temperature adaptability in genotypes has primarily relied on screening nurseries, field performance, and visual evaluations focused on survival [45]. Interactions that are difficult to control complicate the separation of water, heat, and biotic factors from germination potential. Consequently, an efficient, reliable, and straightforward approach is necessary to evaluate numerous genotypes for thermotolerance in controlled environments [45]. Numerous studies have employed parameters such as seed germination rate and potential to assess the tolerance of crop genotypes to abiotic stresses across various species [40,42,46]. This evaluation encompasses both vegetative and reproductive, as well as physiological and biochemical factors during the germination stage [47]. Multiple studies made use of the cumulative temperature response index (CTRI) [40,[48], [49], [50]] to categorize different crop genotypes into tolerance groups for temperature stresses.

The study aimed to (a) quantify the impact of temperature on carinata maximum seed germination (MSG) and seed germination rate (SGR), (b) identify the cardinal temperatures for MSG and SGR, and (c) screen carinata genotypes for temperature tolerance.

## 2 Materials and methods

### 2.1 Seed material

The study evaluated eleven advanced carinata genotypes, including three breed types: inbred, double haploid, and hybrid, alongside one commercial check entry, Avanza 641(Table 1). The seeds were collected in Florida (8 genotypes) and Canada (4 genotypes) from Agrisoma Biosciences Inc., Canada (now Nuseed). Prior to the experiment, the seeds were treated with Helix Vibrance, consisting of fungicides (difenoconazole, metalaxyl-M, fludioxonil, and sedaxane) and insecticides (difenoconazole and metalaxyl thiamethoxam). All the seeds were stored in a refrigerator at 4 °C for equal time to maintain optimum quality until time for further use. The seeds with uniform size, shape, and maturity were randomly selected from a single seed lot for each genotype to study the germination behavior of *B. carinata* genotypes across a range of temperatures. The seed weight (SWGT) of individual genotypes was recorded before starting the temperature treatments and was expressed in mg seed^−1^.

**Table 1:** Type and origin of *Brassica carinata* genotypes sourced from Agrisoma Biosciences, 2019 (now Nuseed).

Genotype	Type^a^	Justification
AX17001	Inbred	Selection from SE16-17 AYT (Avanza family selection) Florida
AX17002	Inbred	Selection from SE16-17 AYT (Avanza family selection) Florida
AX17004	Inbred	High shatter tolerance family, good potential in a winter environment
AX17005	Inbred	High shatter tolerance family, good potential in a winter environment
AX17006	Inbred	High shatter tolerance family, good potential in a winter environment
AX17007	Double haploid	Among the highest Sclerotinia incidence, Jay and Quincy, FL
AX17008	Double haploid	Selection from SE16-17 PYTB Florida
AX17009	Double haploid	Selection from SE16-17 PYTA Florida
AX17010	Double haploid	Selection from SE16-17 PYTB Florida
AX17014	Hybrid	Top 2016-17 Quincy test hybrid Florida
AX17015	Hybrid	Promising test hybrid from 2017, frost-tolerant female
Avanza 641	Check	Commercial check

a Genotypes are classified into three types (I = inbred, DH = double haploid, and H = hybrid). Seed trials (SE - Southeast, AYT - advanced yield trial, PYT - preliminary yield trial).

### 2.2 Seed germination and temperature treatments

The germination of carinata seeds was evaluated under various temperatures from May to September 2019. The study was conducted *in vitro* at the Environmental Plant Physiology Laboratory, Mississippi State University, Mississippi State, MS, USA. The study was performed following the guidelines established by the Association of Official Seed Analysts [51,52] without humidity control. The experiment followed a completely randomized, two-factorial design with genotypes and temperatures serving as the primary source of variation. A total of 12 carinata genotypes were subjected to eight temperature treatments, with each treatment replicated four times using 100 seeds per replication. The eight levels of germination temperatures ranged from 8 to 37 °C in 5 °C increments; however, the actual temperatures recorded during the experiment were 8.2, 12.73, 15.65, 19.93, 23.8, 29.28, 34.22, and 36.96 °C. Each experimental unit consisted of 100 counted and weighed seeds, which were uniformly arranged on sterilized plastic trays measuring 31 cm in length × 24 cm in width. The trays were lined with double layers of sterilized paper towels (Scott Shop towels, Kimberly-Clark, Irving, TX, USA). Paper towels were wetted with sterile distilled water, and trays were covered to reduce moisture loss and arranged vertically in a germination chamber (Fisher Scientific, Inc., Suwanee, GA, USA), maintained at designated treatment temperatures. The chamber's internal temperature was measured using data loggers (WatchDog Model 100, Spectrum Technologies, Inc., Aurora, IL, USA) positioned uniformly on the top, middle, and bottom shelves. After incubation, the trays were examined at 2-h intervals to record seed germination. Seeds were counted as germinated when the radicle reached a length of at least 50 % of the seed's total length. The germinated seeds were counted, recorded, and subsequently discarded. The seed germination experiment was concluded after observing no germination for five consecutive days or eight days post-incubation.

### 2.3 Germination-time course curve fitting procedure

Temperature and seed germination time course data were analyzed using a 4-parameter Weibull function (Eq. (1)) in Sigma Plot 13 (Systat Software, Inc., San Jose, CA, USA).

(Eq.1) $$Y=a*\;\left[1-e^{-{\left[\frac{x-x_{0}+b{\ln\;2}^{\frac{1}{c}}}{b}\right]}^{c}}\right]$$

This function estimates the total seed germination percentage (Y) using the maximum cumulative seed germination percentage (a) at a specific time (x), the shape (c) and steepness (b) of the curve, and the time required to achieve 50 % of the MSG (x_0_). The reciprocal of the time to 50 % of the cumulative MSG (x_0_) was used as the rate of development or the seed germination rate (SGR).

### 2.4 Determination of cardinal temperatures

The responses of MSG and SGR to temperature treatments were analyzed using best-fitted linear and nonlinear regression models to ascertain the cardinal temperatures for all tested genotypes. The optimal curve fitting model was determined based on the highest coefficient of determination (*r*^2^) value. A quadratic model effectively described the relationship between MSG and SGR response to temperature, evidenced by a mean r^2^ of 0.85. In contrast, the modified bilinear model test resulted in an overestimation of T_max_ and an underestimation of T_min_ for SGR. The quadratic model was employed to estimate MSG (%; Eq. (2)) and SGR (d^−1^; Eq. (3)). The cardinal temperatures (T_min_, T_opt_, and T_max_) were determined using Equations (Eq. 4), (Eq. 5), (Eq. 6)). The temperature adaptability range (TAR) for each genotype was determined using Equation (7).

(Eq. 2) $$MSG=a+bT+{cT}^{2}$$

(Eq. 3) $$SGR=a+bT-{cT}^{2}$$

(Eq. 4) $$T_{opt}=\frac{-b}{\left(2c\right)}$$

(Eq. 5) $$T_{\min}=\frac{-b+\sqrt{b^{2}-4ac}}{2c}$$

(Eq. 6) $$T_{\max}=\frac{-b-\sqrt{b^{2}-4ac}}{2c}$$

Where:

T_min_, T_opt_, and T_max_ are the minimum, optimum, and maximum cardinal temperatures for seed germination.

*a*, *b*, and *c* are genotype-specific regression constants.

T is the treatment temperature at which MSG was determined.

(Eq. 7) $$TAR=T_{\max}-T_{\min}$$

### 2.5 Cumulative high-temperature response index (CHTRI)

The high-temperature response indices were computed based on the standardized vigor index [4,53]. The individual temperature response index (ITRI) for each of the six parameters (P), specifically, MSG, SGR, T_opt_, and T_max_ of MSG and SGR, was assessed for high-temperature tolerance across each genotype. The computation involved dividing the value for each genotype by the maximum value observed across all studied genotypes. The cumulative high-temperature response index (CHTRI) was determined by summing the six ITRI derived from MSG, SGR, and T_opt_, and T_max_ of MSG and SGR.

### 2.6 Cumulative low-temperature response index (CLTRI)

The ITRI for low-temperature tolerance was derived from MSG, SGR, T_min_ and T_opt_ temperatures for MSG and SGR, which are analogous to the determination of CHTRI. The ITRI from T_min_ from MSG and SGR was calculated by dividing the minimum value by the values for each genotype observed across all the genotypes. The cumulative low-temperature response index(CLTRI) was determined by summing all six ITRI values.

### 2.7 Data analyses

Regression procedures in Sigma Plot 13 were used to estimate MSG with time and to fit Weibull and polynomial functions for cumulative time series and germination rate data. The MSG, SGR, and SWGT data were analyzed using two-way factorial ANOVA and the ‘doebioresearch’ package in R studio. The data for T_min,_ T_opt_, T_max_, and TAR were analyzed using the PROC GLM (one-way ANOVA) procedure in SAS to determine the effect of the germination temperature treatments on MSG and SGR together with their respective cardinal temperatures. Means were separated using Fisher's protected least significant difference (LSD) at *p* < 0.05. Temperature and time to germination were considered as independent variables, while germination factors (MSG and SGR) were dependent variables.

#### 2.7.1 Principal component analysis (PCA)

The ITRI for each of the six parameters, *i.e*., MSG, SGR, and T_min_, T_opt_, and T_max_ of MSG and SGR of individual genotypes from high- and low-temperature tolerance response index were used to obtain the principal components (Dimension; Dim.) for high- and low-temperature response. The factor vectors were identified using the loadings of each trait at individual principal components. Depending on the genotype loading values, a scatter plot was created for high- and low-temperature tolerance. The analysis was performed in R studio using ‘factoextra’ package [54].

#### 2.7.2 Bubble plot

We utilized the computed CHTRI and CLTRI values to generate a bubble plot aimed at identifying the most high and low-temperature tolerant individual among the carinata genotypes under study. The ‘ggplot2’ package in R studio created the bubble plot.

## 3 Results and discussion

Temperature is a critical factor in the regulation of crop growth and development. This study is the first attempt to address the impact of temperature on seed germination traits across multiple *B. carinata* genotypes. This functional algorithm between seed germination and temperature derived in the study will be instrumental in developing models for carinata in field applications. The thermotolerance capacity observed among carinata genotypes will assist breeders in developing new germplasm suitable for both low and elevated temperatures during seed germination.

### 3.1 Germination time course

**Fig. 1:**
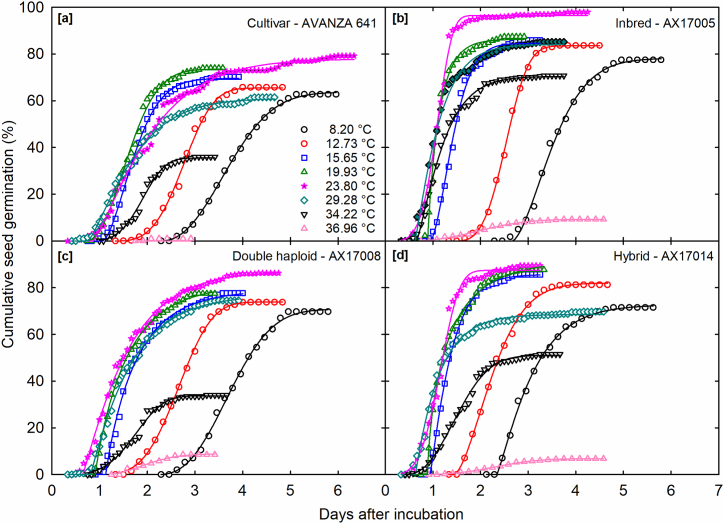
Seed germination time courses of four *Brassica carinata* genotypes representing various groups, (a) cultivar – Avanza 641, (b) Inbred – AX17005, (c) Double haploid – AX17008, and (d) Hybrid – AX17014, germinated at eight temperatures (8.2, 12.73, 15.65, 19.93, 23.8, 29.28, 34.22, and 36.96 °C); the symbols indicate the observed cumulative germination data, and the lines indicate the germination time courses fitted using a 4-parameter Weibull function. Data are means of four replications.

**Fig. 2:**
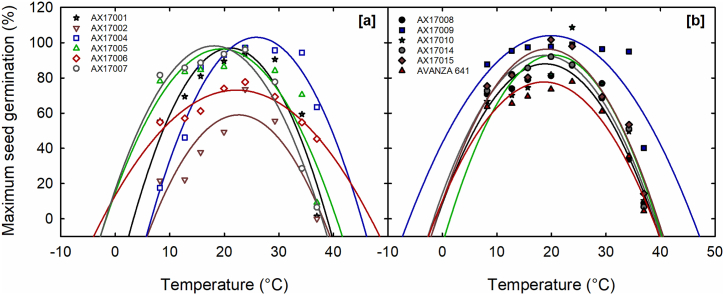
Temperature effects on maximum seed germination (MSG) of 12 *Brassica carinata* genotypes. (a) Genotypes, AX17001, AX17002, AX17004, AX17005, AX17006, and AX17007, (b) AX17008, AX17009, AX17010, AX17014, AX17015, and Avanza 641. The lines are fitted with quadratic equations. Data are means of four replications. The symbols observed the maximum seed germination percentage.

The impact of temperature on cumulative MSG and SGR, as well as variations in germination time, was examined across all carinata genotypes (Supplementary Fig. 1). The impact on four different breed types, viz., cultivar (Fig. 1a), inbred (Fig. 1b), double haploid (Fig. 1c), and hybrid (Fig. 1d), are shown in Fig. 1. A four-parameter Weibull function exhibited a good fit across all tested carinata genotypes (Table 2; mean r^2^ = 0.86). All genotypes exhibited variability in their response to various temperature treatments. At the maximum temperature tested (36.96 °C), fewer than 10 % of germination was recorded for over 50 % of the genotypes. The cumulative MSG for all genotypes declined below 23.80 °C. The MSG was highest (100 %; AX17010) at 23.80 °C and lowest (0 %; AX17002) at 36.96 °C (Fig. 2a and b). Dawadi et al. [1] indicated that the carinata seed MSG exceeded 80 % at a constant temperature of 25 °C, taking approximately 60 h to achieve this MSG, with germination onset occurring after 12 h. The duration required for the initiation of germination (≤24 h) exhibited variability among carinata genotypes and treatments in this study (Fig. 1). Seed emergence occurred most rapidly at temperatures of 23.80 °C and 29.28 °C, within 0.5 days. The two lower temperatures (8.20 and 12.73 °C) and the highest temperatures (36.96 °C) resulted in a delay of seed emergence exceeding one day.

**Table 2:** Quadratic equation constants (a, b, and c), coefficients of determination (*r*^2^), cardinal temperatures (T_min_, T_opt_, and T_max_), maximum seed germination (MSG), temperature adaptability range (TAR) for MSG, and mean individual seed weight (SWGT) of 12 *Brassica carinata* genotypes evaluated under eight temperature treatments.

Genotype		Equation constants			*r*^2^	Cardinal temperatures (°C)			TAR (°C) d	SWGT (mg seed^−1^)^c^
	MSG (%)^c^	a	b	c		T_min_^d^	T_opt_^d^	T_max_^d^		
AX17001	96.97^bc^	−39.76	12.96	−0.3073	0.85	3.33^bc^	21.09^c^	38.86^b^	35.53^cd^	0.426^e^
AX17002	59.01^f^	−66.19	11.08	−0.2452	0.77	7.08^a^	22.60^b^	38.11^b^	31.03^d^	0.372^f^
AX17004	100.0^a^	−82.47	14.34	−0.2770	0.93	6.59^ab^	25.89^a^	45.18^a^	38.59^b^	0.343^g^
AX17005	96.48^b^	14.88	8.39	−0.2159	0.76	−1.70^d^	19.44^de^	40.58^b^	42.28^b^	0.506^ab^
AX17006	73.12^e^	13.32	5.38	−0.1208	0.84	−2.35^cd^	22.25^b^	46.85^a^	49.21^ab^	0.378^f^
AX17007	98.20^b^	16.61	9.03	−0.2498	0.95	−1.75^d^	18.07^f^	37.90^b^	39.65^c^	0.500^b^
AX17008	88.00^ed^	8.56	8.43	−0.2237	0.92	−0.99^d^	18.85^f^	38.68^b^	39.67^c^	0.408^e^
AX17009	100.0^a^	43.09	6.12	−0.1536	0.64	−6.11^e^	19.92^d^	45.94^a^	52.05^a^	0.514^a^
AX17010	93.00^c^	−14.53	10.58	−0.2601	0.82	−1.42^cd^	20.33^d^	39.24^b^	37.82^c^	0.384^ef^
AX17014	92.8^c^	14.10	8.42	−0.2254	0.94	−1.60^d^	18.69^ef^	38.98^b^	40.58^c^	0.493^bc^
AX17015	96.37^bc^	9.70	9.02	−0.2346	0.91	−1.05^d^	19.22^d^	39.49^b^	40.54^c^	0.470^d^
AVANZA641	77.71^e^	10.04	7.27	−0.1952	0.93	−1.33^d^	18.62^f^	38.57^b^	39.91^c^	0.428^e^
Mean	89.30	˗	˗	˗	0.86	−0.14	20.41	40.70	40.57	0.4355
LSD	4.7^b^	˗	˗	˗	˗	4.43^b^	0.89^b^	3.74^b^	7.97^a^	0.0203^b^

The letters near the values are the results of Fisher's protected least significant difference (LSD); values with different letters are significantly different (p < 0.05), and the same letters are not significantly different (p > 0.05).

a Significant at the 0.05 probability level.

b Significant at the 0.001 probability level.

c Two-way ANOVA was performed, taking temperature and genotype as main factors. The effect of temperature, genotype, and interaction were significant at p < 0.001 for MSG and SWGT.

d One-way ANOVA was performed, taking genotype as the main factor.

Temperature significantly influences seed germination [23,24,55]. The available data on the germination capacity and rate of carinata across various temperatures is limited. This study established a functional database for temperature and seed germination traits, as well as a thermotolerance classification for carinata genotypes.

### 3.2 Maximum seed germination response to temperature

A significant genotype × temperature interaction was observed on MSG (p < 0.001). The response of MSG to temperature was modeled using a quadratic regression, yielding a mean *r*^2^ of 0.86. The response of carinata genotypes to temperature for MSG exhibited variability, with values ranging from 59.01 % (AX17002) to 100 % (AX17004 and AX17009), resulting in a mean of 89.30 % across genotypes (Table 2; Fig. 2a and b). The variations in response patterns contributed to the interaction effect.

Parameters derived from the germination-time course data were used to calculate T_min_, T_opt_, and T_max_ for MSG. The cardinal temperatures varied significantly among (*p* < 0.001; Table 2). The estimated T_min_ values varied from −6.11 (AX17009) to 7.08 °C (AX17002), yielding a mean of −0.14 °C. The mean T_opt_ was 20.41 °C. Genotype AX17004 exhibited the highest T_opt_ value at 25.89 °C, whereas genotype AX17007 recorded the lowest at 18.07 °C. The mean T_max_ was 40.70 °C, with genotype AX17007 exhibiting the minimum T_max_ value of 37.90 °C, whereas genotype AX17006 and AX17009 displayed the maximum values of 46.85 and 45.94 °C which are statistically non-significant (Table 2). The TAR (T_max_-T_min_) indicated genotype-specific germination capacities across different temperature conditions. Carinata genotypes exhibited variations in TAR (p < 0.05; Table 2), with a mean value of 40.57 °C, ranging from 31.03 °C (AX17002) to 52.05 °C (AX17009).

Research on the germination response to temperature in various plant species indicates that while there is variability in seed germination, utilizing this parameter for genotype screening may be impractical due to the influence of multiple factors on MSG [39]. These factors include seed traits [56], the duration of storage between ripening and planting [57], and the environmental conditions during maternal seed production [58]. The variation in TAR among genotypes suggests that differing genetic traits influence their adaptation and performance under varying temperatures [21]. The optimal temperatures for MSG differed among genotypes, exhibiting a range of responses with varying MSG levels across all genotypes. Genotype AX17002 exhibited the lowest percentage of MSG at 59.01 %. The cardinal temperatures for canola, as indicated by a comparable model, show a T_min_ range of 0 to 3 °C, T_opt_ of 29 to 33 °C, and T_max_ of 35 °C [41]. Our study indicated that the values for T_min_ and T_opt_ ranges were lower, whereas T_max_ was higher. In Canada and the northern tier states of the United States, carinata and canola are grown in similar areas. Establishing the cardinal temperatures for carinata genotypes aids plant breeders and producers by enhancing the understanding of crop species and their necessary conditions for establishment.

### 3.3 Seed germination rate response to temperature

**Fig. 3:**
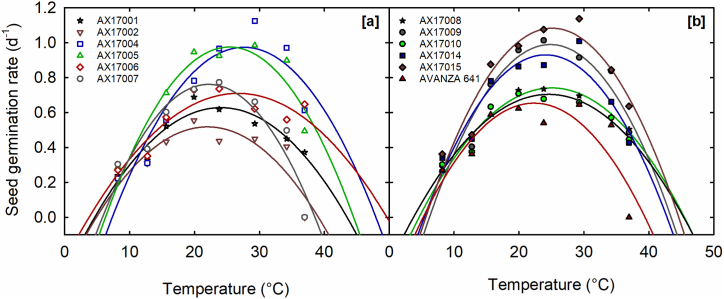
Temperature effects on seed germination rate (SGR) of 12 *Brassica carinata* genotypes. (a) Genotypes, AX17001, AX17002, AX17004, AX17005, AX17006, and AX17007, (b) AX17008, AX17009, AX17010, AX17014, AX17015, and Avanza 641. Data are means of four replications. The symbols are observed seed germination rates.

A quadratic regression model effectively characterized the relationship between SGR and temperature (mean r^2^ = 0.85; Table 3). A significant genotype × temperature interaction was observed on SGR (p < 0.001). Among genotypes, SGR increased with a temperature rise from 8.2 to 23.8 °C, followed by a gradual decline as the temperature reached 36.69 °C. The estimated SGR was highest at 0.98 d^−1^ at 19.93 °C and lowest at 0 d^−1^ at 36.96 °C (Fig. 3a and b). The findings align with similar results obtained from eight rapeseed cultivars assessed within a temperature range of 3 to 23 °C [59].

**Table 3:** Quadratic equation constants (a, b, and c), coefficients of determination (*r*^2^), cardinal temperatures (T_min_, T_op_t, and T_max_), seed germination rate (SGR), and temperature adaptability range (TAR) for SGR, of 12 *Brassica carinata* genotypes evaluated under eight temperature treatments.

Genotypes		Equation constants				Cardinal temperatures (°C)			
	SGR (d^−1^)^c^	a	b	c	*r*^2^	T_min_^d^	T_opt_^d^	T_max_^d^	TAR^d^
AX17001	0.63^f^	−0.3342	0.0799	−0.0017	0.88	4.63^c^	22.04^c^	43.45^ab^	38.82^bc^
AX17002	0.52^g^	−0.339	0.0779	−0.0018	0.75	4.90^c^	21.98^d^	39.06^c^	34.17^c^
AX17004	0.97^b^	−0.8306	0.1305	−0.0024	0.85	7.34^a^	27.65^a^	47.96^a^	40.62^a^
AX17005	0.97^b^	−0.7459	0.1355	−0.0027	0.86	6.28^a^	25.38^b^	44.49^ab^	38.20^bc^
AX17006	0.71^e^	−0.2518	0.072	−0.0013	0.82	3.76^d^	26.71^ab^	49.65^a^	45.89^a^
AX17007	0.76^d^	−0.6386	0.1261	−0.0028	0.85	5.83^abc^	22.20^c^	38.57^c^	32.74^c^
AX17008	0.70^e^	−0.2767	0.0798	−0.0016	0.89	3.75^d^	24.55^b^	45.35^ab^	41.60^a^
AX17009	0.99^b^	−0.7696	0.1417	−0.0029	0.89	6.21^a^	24.82^b^	43.42^b^	37.22^bc^
AX17010	0.74^d^	−0.3717	0.0888	−0.0018	0.93	4.61^c^	25.08^b^	45.56^ab^	40.95^ab^
AX17014	0.93^c^	−0.6156	0.1283	−0.0027	0.86	5.41^abc^	24.11^b^	42.81^b^	37.41^bc^
AX17015	1.08^a^	−0.7178	0.1433	−0.0029	0.90	5.64^abc^	25.14^b^	44.64^ab^	39.00^bc^
AVANZA 641	0.66^f^	−0.4614	0.0999	−0.0022	0.71	5.23^b^	22.40^b^	39.57^c^	34.34^c^
Mean	0.81	˗	˗	˗	0.85	5.30	24.51	43.71	38.41
LSD	0.04^b^	˗	˗	˗	˗	2.03^a^	1.73^b^	4.58^a^	6.58^b^

The letters near the values are the results of Fisher's protected least significant difference (LSD); values with different letters are significantly different (p < 0.05), and the same letters are not significantly different (p > 0.05).

a Significant at the 0.05 probability level.

b Significant at the 0.001 probability level.

c Two-way ANOVA was performed, taking temperature and genotype as main factors. The effect of temperature, genotype, and interaction were significant at p < 0.001 for SGR.

d One-way ANOVA was performed, taking genotype as the main factor.

The cardinal temperatures for SGR varied significantly across the genotypes (*p* < 0.05; Table 3). The T_min_ ranged from 3.75 °C (AX17008) to 7.34 °C (AX17004), with a average of 5.30 °C. The estimated mean of T_opt_ was 24.51 °C, with a range from 21.98 °C (AX17002) to 27.65 °C (AX17004). The two genotypes, AX17007 and AX17002, exhibited the lowest T_max_ value of 38.57 °C and 39.06 °C, respectively. The highest T_max_ was 49.65 (AX17006), resulting in an estimated mean of 43.71 °C (Table 3).

The TAR for SGR varied between 32.74 °C (AX17007) and 45.89 °C (AX17006) with an average of 38.41 °C (Table 3). Unlike reports on other crop species, the SGR for carinata varied across a range of temperatures, lacking a clearly defined optimum [40,42]. The rate decreased linearly and rapidly with rising temperatures. Soltani et al. [38] reported that in most plant species, the growth rate increases from minimum to optimum temperatures and declines between optimum and maximum temperatures, consistent with the quadratic model predictions and actual data for the carinata genotypes studied. In our study, the SGR cardinal temperatures exceeded those of MSG, consistent with findings on ornamental peppers [39] and switchgrass [40]. This aligns with the findings of Schimpf et al. [60], indicating that the MSG exhibits lower temperature sensitivity compared to SGR. Seed germination is a temperature-dependent process; however, variation in cardinal temperatures among genotypes exists due to intra-specific differences related to genetic diversity, area of origin, or adaptation of these entries. Comparable results were observed for switchgrass [40]. Kiniry et al. [61] proposed that cardinal temperatures may vary based on genotype and specific processes.

### 3.4 Principal component analysis (PCA) for high-temperature tolerance

**Fig. 4:**
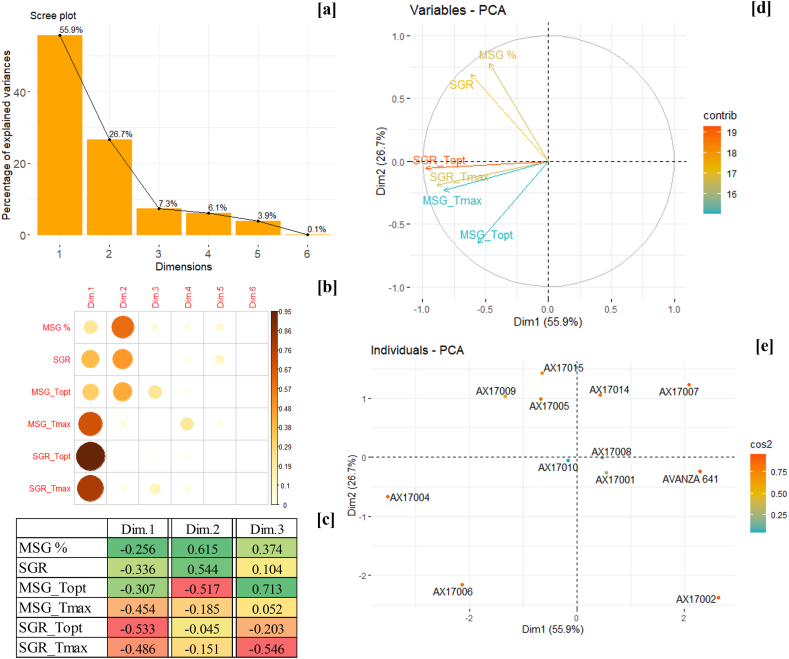
The principal component analysis of 12 carinata genotypes for high-temperature tolerance, (a) Scree plot of six principal components with their respective variance (%), (b) Correlations of principal components with the variables (MSG, SGR, and T_opt,_ and T_max_ of both MSG and SGR), and (c) The individual parameter loadings towards the principal components, Dim.1 and Dim.2. The red to green color indicates the least to highest loading values of parameters in each dimension, (d) Variables as factor vectors at first two principal components (Dim.1 and Dim.2), and (e) Scatter plot of 12 carinata genotypes on the Dim.1 and Dim.2 factor planes based on the factor scores.

The PCA is an efficient dimension reduction technique widely used in data analysis [62]. It simplifies the data into a set of uncorrelated variables called principal components (Dim.) based on the original data points. The method has been extensively used in the screening and selection of varieties and hybrids in multiple crops [63,64]. The PCA analysis of carinata genotypes using six parameters (MSG, SGR, T_opt,_ and T_max_ of both MSG and SGR) resulted in six dimensions (Dim) with a major contribution from Dim.1 (55.9 %) and Dim.2 (26.7 %) (Fig. 4a). The correlation between parameters and Dim.s suggests that T_opt_ of SGR (r^2^ = 0.90) is highly contributing for Dim.1, followed by T_max_ for SGR (r^2^ = 0.77), T_max_ for MSG (r^2^ = 0.65), and SGR (r^2^ = 0.59) (Fig. 4b). On the other hand, Dim.2 was highly explained by MSG (r^2^ = 0.59). The fourth, fifth, and sixth Dimensions were only slightly correlated with the tested parameters from the studied genotypes.

Depending on the loadings on Dim.1 and Dim.2, the parameters were placed on the vector planes (Fig. 4c and d). The Dim.1 was negatively related to all the high-temperature tolerance parameters, featuring the first quadrant as a low-temperature tolerant plane. The MSG and SGR had positive loadings on Dim. 2, while the other parameters were negatively associated with Dim.2 (Fig. 4c and d). The T_max_ for MSG and SGR was located in the third factor quadrant, indicating that the genotypes placed in this quadrant will be tolerant to high temperatures. In addition, the MSG and SGR were placed in the second quadrant, indicating this will accommodate genotypes with high-temperature tolerance. Meanwhile, the first and fourth quadrants harbor high-temperature sensitive genotypes (Fig. 4d).

Based on the loadings of the carinata genotypes, a scatter plot was created revealing their temperature tolerance levels (Fig. 4e). The genotype AX17004 was the most high-temperature tolerant one due to its high loading value in the third quadrant, followed by AX17006. In the second quadrant, genotype AX17009 had the highest loading, showing its high-temperature tolerance. The second also accommodates AX17005 and AX17015 genotypes, expressing their moderate high-temperature tolerance compared to other genotypes. However, AX17007 and AX17002 were located in the first and fourth quadrants with a high loading value, revealing their notable high-temperature sensitivity among all the studied genotypes. The Avanza 641 was also located in the fourth quadrant, expressing its high-temperature sensitivity along with other sensitive genotypes.

### 3.5 Principal component analysis for low-temperature tolerance

**Fig. 5:**
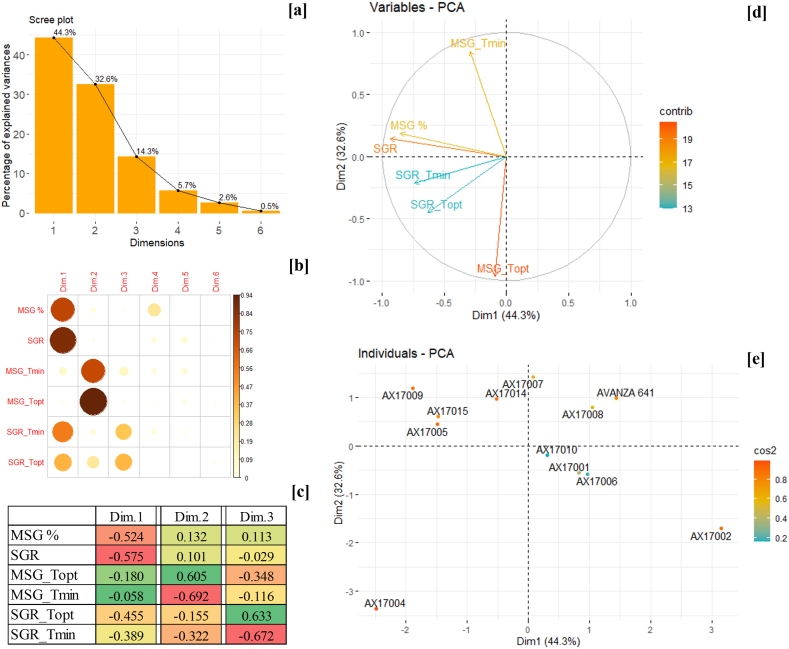
The principal component analysis of 12 carinata genotypes for low-temperature tolerance, (a) Scree plot of six principal components with their respective variance (%), (b) Correlations of principal components with the variables (MSG, SGR, and T_opt,_ and T_min_ of both MSG and SGR), and (c) The individual parameter loadings towards the principal components, Dim.1 and Dim.2. The red to green color indicates the least to highest loading values of parameters in each dimensions, (d) Variables as factor vectors at first two principal components (Dim.1 and Dim.2), and (e) Scatter plot of 12 carinata genotypes on the Dim.1 and Dim.2 factor planes based on the factor scores.

As with the high-temperature tolerance, the PCA was performed for low-temperature tolerance using six factors derived from the seed germination assay (MSG, SGR, T_min,_ and T_opt_ for both MSG and SGR), where each parameter varied in their input based on its relation to the maximum or minimum constant for that particular factor measured across all the genotypes. The PCA analysis generated six principal components (Dim.s) to explain 100 % variance among the genotypes tested. The first two components collectively accounted for 76.9 % of the total variance (Fig. 5a). The SGR was highly correlated with Dim.1 (r^2^ = 0.90), followed by MSG (r^2^ = 0.71) (Fig. 5b). While Dim.2 was highly correlated with T_opt_ for MSG (r^2^ = 0.92). All the parameters were negatively associated with Dim.1 (Fig. 5c). In contrast, Dim.2 was positively associated with MSG, SGR, and T_opt_ for MSG (Fig. 5c). This contributed to the separation of the parameters in the four-factor quadrants (Fig. 5d). The Tmin for MSG and SGR was located in the second and third quadrant, respectively. Thus, genotypes in this quadrant will have low-temperature tolerance.

The first and fourth quadrants accommodated genotypes such as Avanza 641 and AX17002 with low-temperature sensitivity. From the scatter plot using Dim.1 and Dim.2 components, the genotype AX17009 was observed to have a better low-temperature tolerance capacity, followed by AX17005 and AX17015. However, the genotypes harboring in the third quadrant (AX17004) exhibited a profile of being moderately low-temperature tolerant. A crucial goal for most plant breeding programs is to identify genotypes with essential traits that make them resilient to extreme thermal conditions [47], as well as to develop new genotypes with high field stress tolerance that can thrive under variable weather conditions [65]. Several studies have used identical parameters to screen crops for temperature tolerance [39,40,47]. Evaluating a crop genotype's thermal adaptability range is typically limited to visual field observation, field performance, and nursery screening. In these conditions, separating different abiotic and biotic stress factors is challenging. Hence, there is an urgent need for an environment where these stress factors are controlled. Setimela et al. [45] suggested that evaluating the thermal adaptability range has paved the way for developing faster, more reliable, and low-cost methods to screen a large batch of plant materials for thermotolerance characteristics. In our study, genotype AX17009 was grouped as the most low-temperature tolerant. Also, genotypes AX17005, AX17014, and AX17015 appear to have good germination at the low-temperature treatment. The genotypes AX17002 and Avanza 641 were categorized as low-temperature sensitive. Data concerning thermotolerance screening for carinata genotypes and intraspecific variation in the establishment under different regions and thermal conditions are limited in the literature; however, there is a need for further testing.

### 3.6 Cumulative high- and low-temperature tolerance response index

**Fig. 6:**
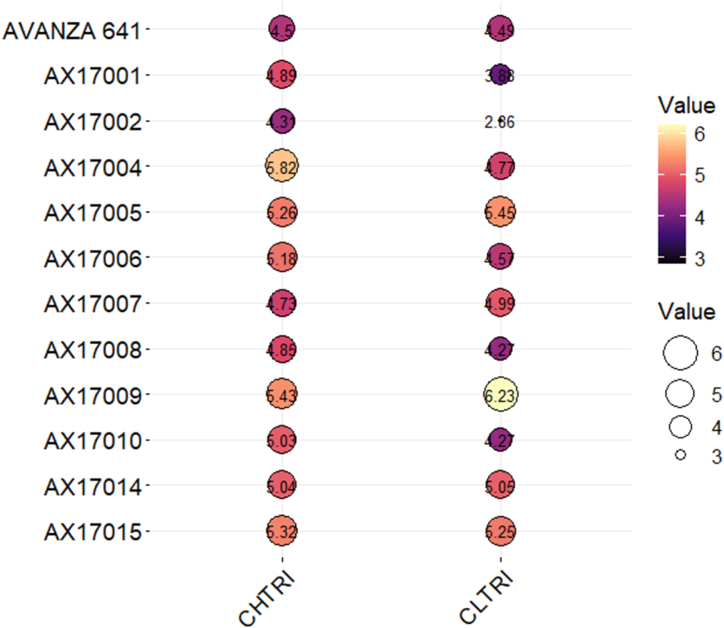
Bubble plot of carinata genotypes for cumulative high-temperature tolerance response index (CHTRI) and cumulative low-temperature tolerance response index (CLTRI).

The cumulative indices for high- and low-temperature tolerance responses were calculated to assess the overall reaction of 12 genotypes based on factors obtained from the germination assay. The CHTRI identified genotypes that are both high-temperature tolerant and sensitive. The genotype AX17004, with a CHTRI value of 5.82, demonstrated significant high-temperature tolerance relative to other genotypes (Fig. 6). The genotypes AX17009 (5.43), AX17015 (5.32), and AX17005 (5.26) exhibited moderate tolerance to high temperatures. Conversely, AX17002 was recognized as a sensitive genotype, exhibiting a CHTRI value of 4.31. In contrast, AX17009 is identified as a low-temperature tolerant genotype, exhibiting a CLTRI value of 6.23. AX17005 achieved this with a value of 5.45, AX17015 at 5.23, and AX17007 at 4.99. The lowest CLTRI values were observed in AX17002 (2.86) and AX 17001 (3.88), indicating their sensitivity to low temperatures relative to the other carinata genotypes examined. The PCA analysis and bubble plot results complement each other, thereby reinforcing the reliability of the identified genotypes for high- and low-temperature tolerance.

### 3.7 Parameter relationships

**Fig. 7:**
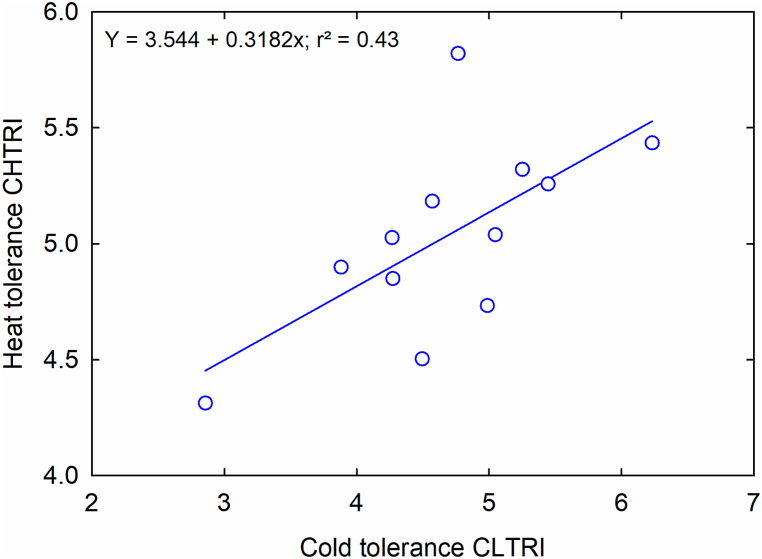
The relationship between cumulative low- and high-temperature response index (CLTRI; CHTRI) of 12 *Brassica carinata* genotypes.

A weak positive linear relationship was identified (*r*^2^ = 0.43) between the cumulative low-temperature response index (CLTRI) and the cumulative high-temperature response index (CHTRI) for 12 evaluated carinata genotypes evaluated (Fig. 7). This relationship suggests that low- and high-temperature tolerance responses among these genotypes are distinct traits. Consequently, identifying genotypes exhibiting both low- and high-temperature tolerance is challenging. Thus, when developing tolerant genotypes, selection for low and high-temperature tolerance must be conducted separately.

**Fig. 8:**
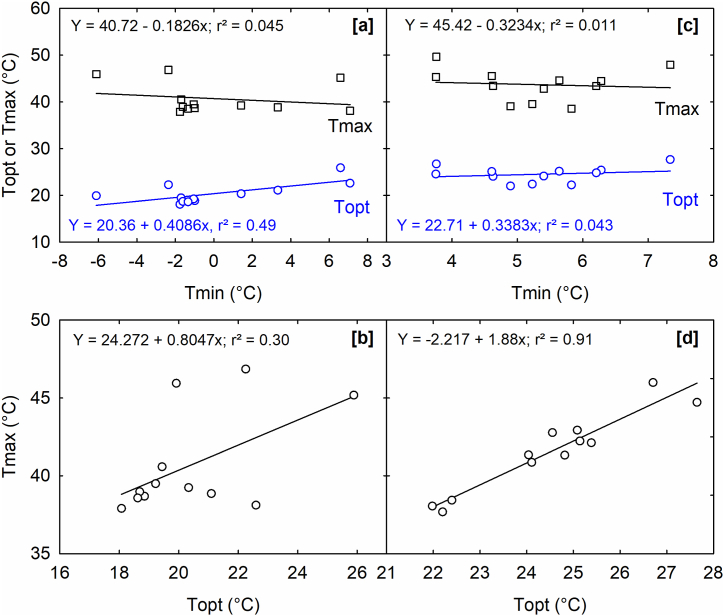
Relationship between the cardinal temperatures for maximum seed germination (MSG) of 12 *Brassica carinata* genotypes, the relationship between (a) minimum (T_min_) and optimum or (T_opt_) and maximum (_Tmax_) temperatures, (b) optimum (T_opt_) and maximum (T_max_) temperatures, and the relationship between (c) seed germination rate (SGR) and minimum (T_min_) and optimum (T_opt_) and maximum (T_max_) temperatures, and (d) seed germination rate optimum (T_opt_) and maximum (_Tmax_) temperatures of 12 *Brassica carinata* genotypes.

A weak linear relationship exists between MSG T_opt_ and T_max_ (r^2^ = 0.30; Fig. 8b), whereas a poor inverse relationship is observed between MSG T_min_ and T_max_ (r^2^ = 0.045; Fig. 8a). As the minimum temperature for carinata genotypes increased, the optimal temperature also increased (r^2^ = 0.49; Fig. 8a). Furthermore, SGR T_max_ exhibited an increase corresponding to the rise in T_opt_ (r^2^ = 0.91; Fig. 8d). A weak inverse relationship was observed between SGR T_min_ and T_max_ (r^2^ = 0.011; Fig. 8c), as well as a weak linear relationship between T_min_ and T_opt_ (r^2^ = 0.043) (Fig. 8c). The study revealed that as the optimum temperature increased, the maximum temperature varied across genotypes but showed a weak correlation with T_opt_, suggesting that each genotype exhibited distinct T_max_ values necessary for achieving MSG. There was a weak correlation between T_min_ and T_opt_. As the minimum temperature for MSG increased, the optimum temperature also rose. The optimal temperature for SGR exhibited a linear increase with maximum temperature; however, T_min_, T_max_, and T_opt_ were more specific to genotypes for SGR, as indicated by the observed weak relationships.

**Fig. 9:**
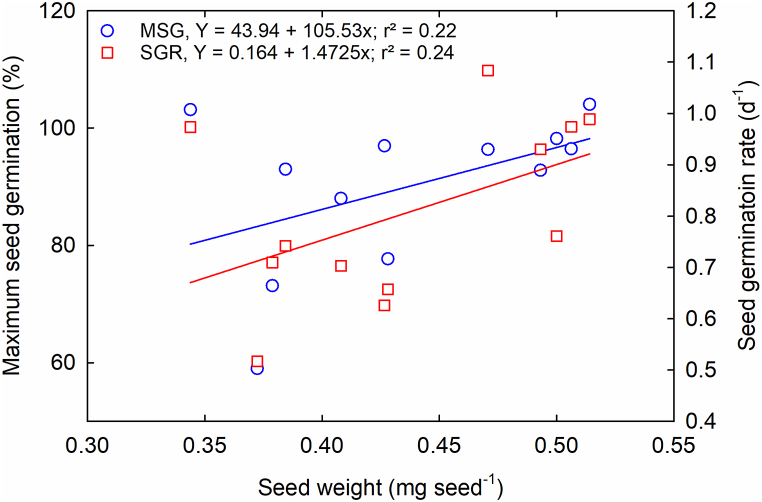
The relationship between maximum seed germination (MSG), seed germination rate (SGR), and seed weight of 12 *Brassica carinata* genotypes.

Research indicates that seed weight was not utilized as a criterion for classifying crop species into thermotolerance groups, owing to variations in origin and the influence of the parental environment [42]. Individual seed weight varied among genotypes (p < 0.001; Table 2) and exhibited a weak correlation with MSG and SGR (Fig. 9), suggesting that seed weight did not influence either MSG or SGR. A weak positive linear relationship was identified between MSG and seed weight (r^2^ = 0.22; Fig. 9) as well as between SGR and seed weight (r^2^ = 0.24; Fig. 9).

This study assessed the impact of temperature on the MSG capacity and SGR of 12 advanced carinata genotypes through *in vitro* analysis. Genotype-specific cardinal temperatures and thermal accumulation rates were estimated using best-fit regression models. The mean minimum, optimum, and maximum temperatures of the MSG for carinata genotypes were −0.14, 20.41, and 40.70 °C, respectively. The mean minimum temperature for SGR was 5.30 °C, the mean optimum temperature was 24.51 °C, and the mean maximum temperature was 43.71 °C. The average TAR for the genotypes was 40.57 °C for MSG and 38.41 °C for SGR. There was a variability among the genotypes concerning MSG, SGR, TAR, and cardinal temperature. Carinata genotypes were categorized into distinct groups based on their seed germination vitality traits, specifically regarding low- and high-temperature tolerance using principal component analysis and temperature response indices. The genotypes AX17004 and AX17009 were identified as the most tolerant to high and low temperatures, respectively. The double haploid and hybrid carinata breeding groups exhibited stable thermotolerance during the seed germination stage.

The inbred group exhibited a more extensive cluster in response to both minimum and maximum temperatures. The research highlighted the necessity of a distinct selection of genotypes exhibiting tolerance to low or high temperatures. The data collected in this study indicate that the identified cardinal temperatures will enhance the development and application of carinata crop models in field production systems. Additionally, identifying genotypes that are tolerant to low and high temperatures will aid in delineating cultivation regions. Future evaluations of these genotypes under field conditions are necessary to identify and quantify those that exhibit tolerance to both low and high temperatures.

## CRediT authorship contribution statement

**Leelawattie Persaud:** Writing – review & editing, Writing – original draft, Visualization, Methodology, Formal analysis. **Naflath Thenveettil:** Writing – review & editing, Formal analysis. **Ramdeo Seepaul:** Writing – review & editing, Funding acquisition. **Bisoondat Macoon:** Writing – review & editing, Supervision, Project administration, Funding acquisition, Formal analysis. **Krishna N. Reddy:** Writing – review & editing. **Kambham Raja Reddy:** Writing – review & editing, Visualization, Supervision, Software, Resources, Project administration, Methodology, Investigation, Funding acquisition, Formal analysis, Data curation, Conceptualization.

## Data availability statement

Data will be made available on request.

## Declaration of competing interest

The authors declare the following financial interests/personal relationships that may be considered potential competing interests: Kambham R Reddy reports that the National Institute of Food and Agriculture provided article publishing charges, equipment, or supplies, and travel.
