# Supplementary material for: Thermotolerance screening of Brassica carinata genotypes using in vitro seed germination assay
Source: Heliyon. 2024 Dec 10;10(24):e41113. doi: 10.1016/j.heliyon.2024.e41113 (PMC11699105; doi:10.1016/j.heliyon.2024.e41113)
Supplement: Multimedia component 1 [file mmc1.docx]

Supplementary Fig. 1. Seed germination time courses for 12 *Brassica carinata* genotypes germinated at a range of temperature (8 – 38°C. The symbols indicate the observed cumulative germination data, and the lines indicate the germination time courses fitted using a 4-parameter Weibull function. Data are means of four replications.
